# Supplementary material for: ERK hyperactivation in epidermal keratinocytes impairs intercellular adhesion and drives Grover disease pathology
Source: JCI Insight. 2024 Nov 8;9(21):e182983. doi: 10.1172/jci.insight.182983 (PMC11601706; doi:10.1172/jci.insight.182983)
Supplement: Supplemental data [file jciinsight-9-182983-s032.pdf]

Supplemental Figure S1

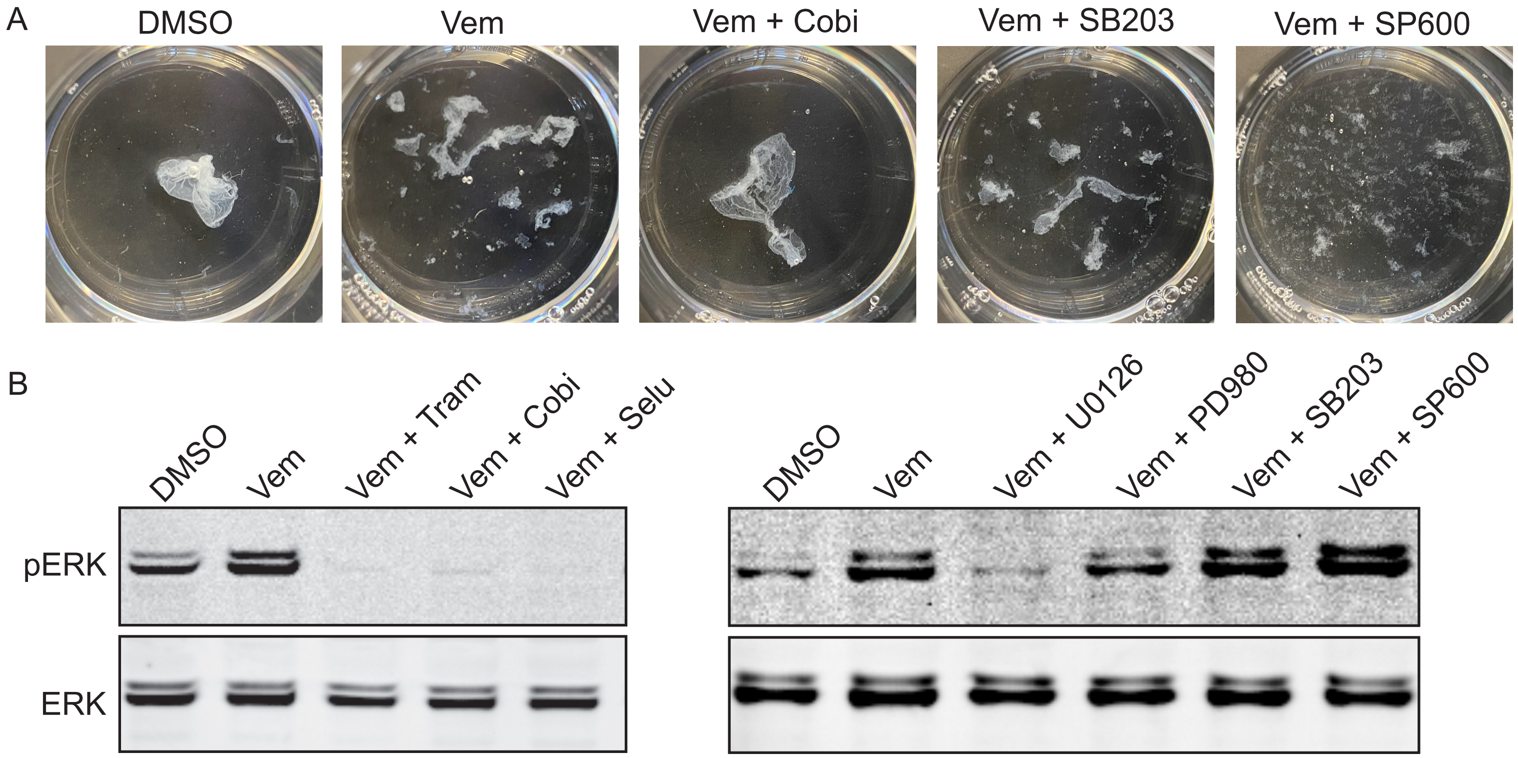

Supplemental Figure S1: MEK inhibition dampens ERK hyper-activation and restores integrity of vemurafenib-treated keratinocyte sheets. (A) Images of drug-treated keratinocyte monolayers after a mechanical dissociation assay; representative images of fragmented cell sheets transferred into 6-well cell culture plates are shown. (B) Immunoblot of total and phosphorylated ERK (pERK) in lysates from NHEKs treated for 24 h with vemurafenib (Vem, 10 µM) +/- MEK inhibitors trametinib (Tram, 1 µM), cobimetinib (Cobi, 1 µM), selumetinib (Selu, 1 µM), U0126 (10 µM), PD98059 (PD980, 20 µM), a p38 inhibitor SB203580 (SB203, 10 µM), or the JNK inhibitor SP600125 (SP600, 25 µM).

Supplemental Figure S2

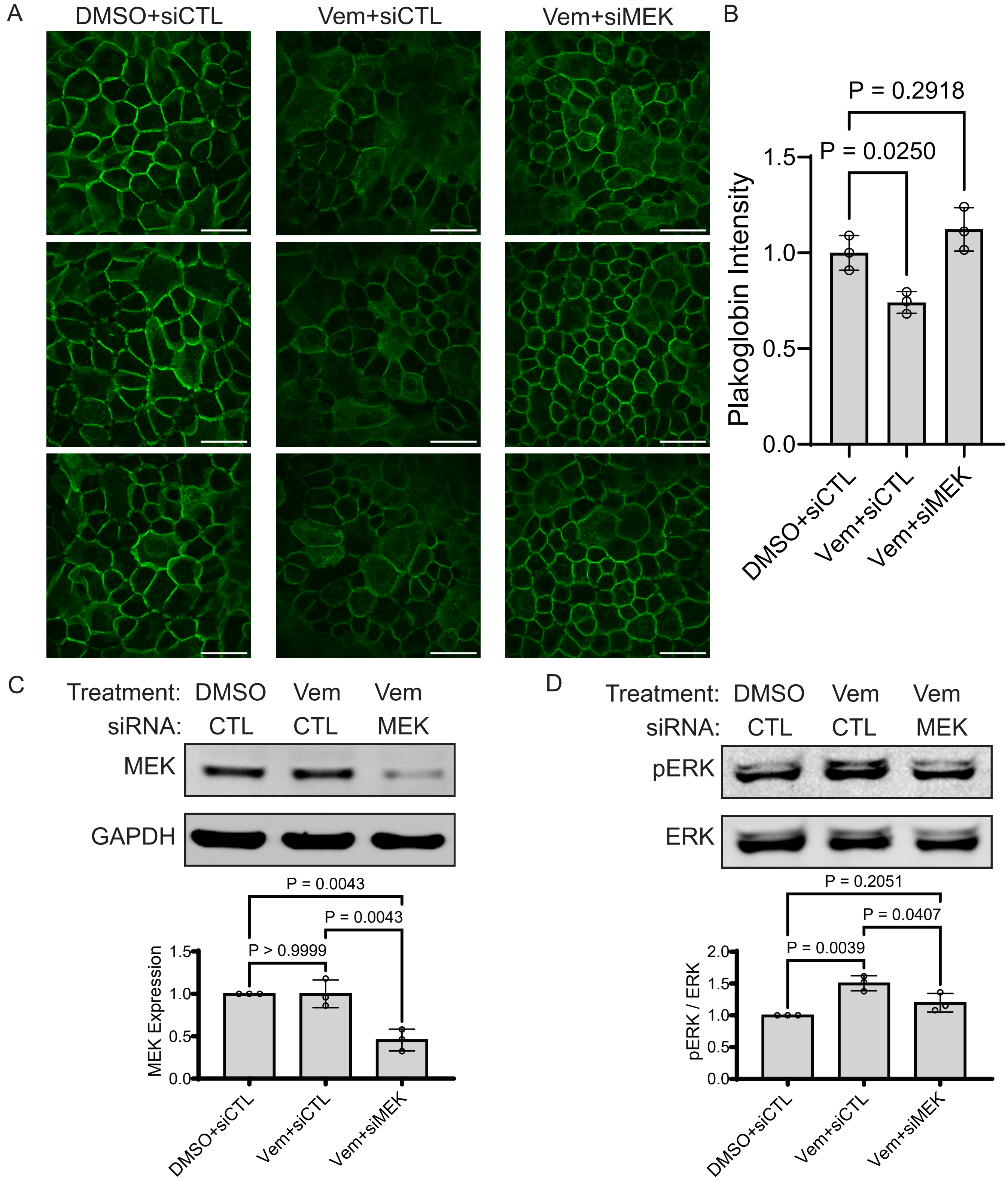

Supplemental Figure S2: Silencing of MEK enhances desmosomal protein localization in vemurafenib-treated keratinocytes. NHEKs were transfected with control siRNA (siCTL) or siRNA against MEK1/2 (siMEK) for 48 hrs then were treated with DMSO or vemurafenib (10 μM) for 24 h prior to fixation or lysis. (A) Confocal immunofluorescence images of plakoglobin; scale bar = 50 μm. (B) Bar graph displays the mean ± SD of normalized plakoglobin intensity (mean of DMSO+siCTL set to 1) from ≥10 images across each treatment group with data points plotted for N=3 experimental replicates. (C) Immunoblot of MEK (GAPDH loading control); bar graph displays the mean ± SD of the ratio of MEK/GAPDH band intensities (DMSO+siCTL set to 1) with data points plotted for N=3 experimental replicates. (D) Immunoblot of pERK and ERK; bar graph displays the mean ± SD of the ratio of pERK/ERK band intensities (DMSO+siCTL set to 1) with data points plotted for N=3 experimental replicates. All P-values are from one-way ANOVA using the Bonferroni adjustment for multiple comparisons.

Supplemental Figure S3

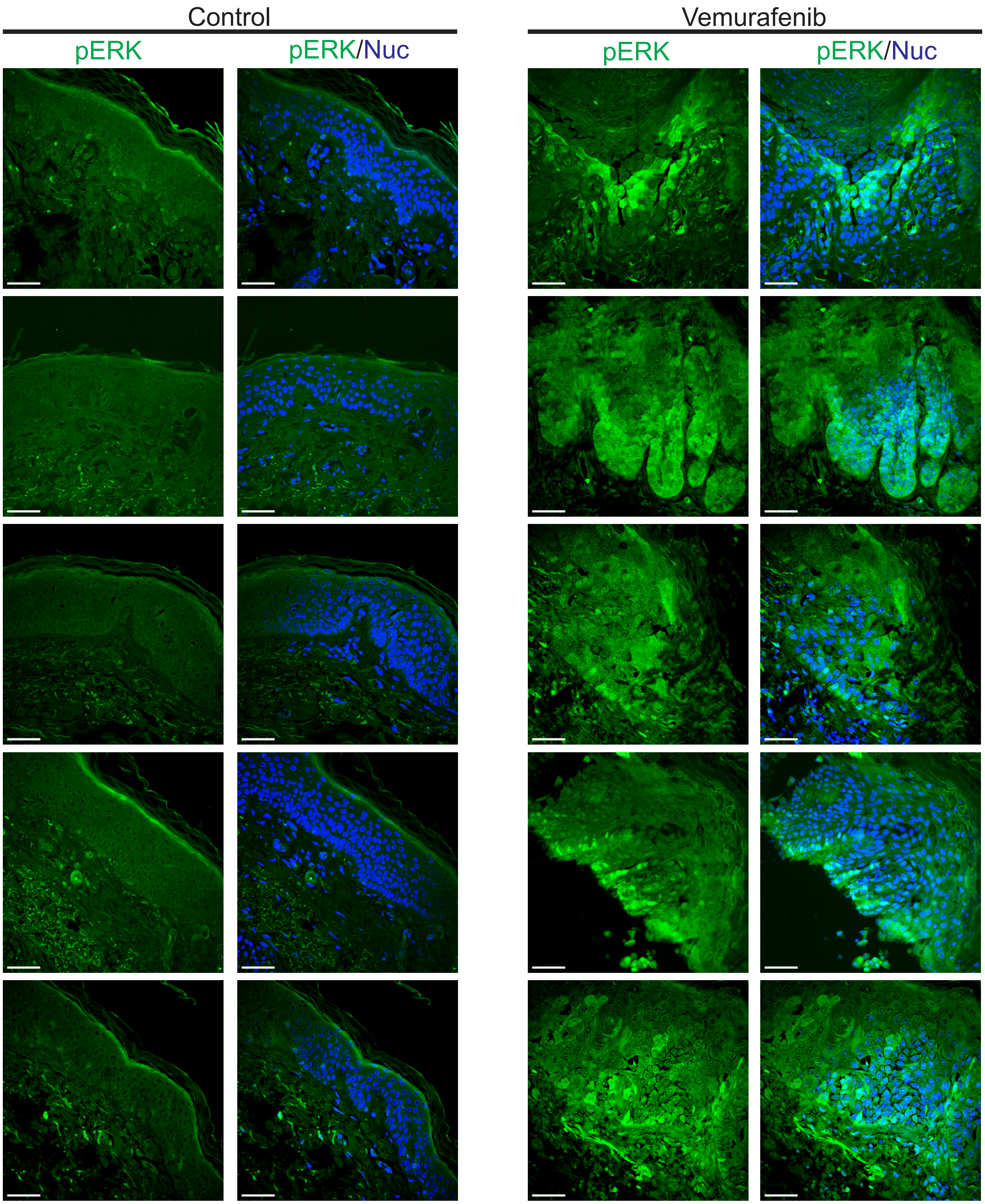

Supplemental Figure S3: Additional biopsies of B-RAF inhibitor-induced Grover disease display epidermal hyperactivation of ERK. Immunostaining of pERK and Hoechst to stain nuclei (Nuc) in tissue cross-sections from patient biopsies; images shown are from 5 control biopsies and from 5 patients with vemurafenib-induced Grover disease; scale bar = 50 μm.
