# Supplementary material for: ERK hyperactivation in epidermal keratinocytes impairs intercellular adhesion and drives Grover disease pathology
Source: JCI Insight. 2024 Nov 8;9(21):e182983. doi: 10.1172/jci.insight.182983 (PMC11601706; doi:10.1172/jci.insight.182983)

Figure 1A: pERK
Rabbit anti-phospho-ERK1/2 (D13.14.4E)
Cell Signaling #4370

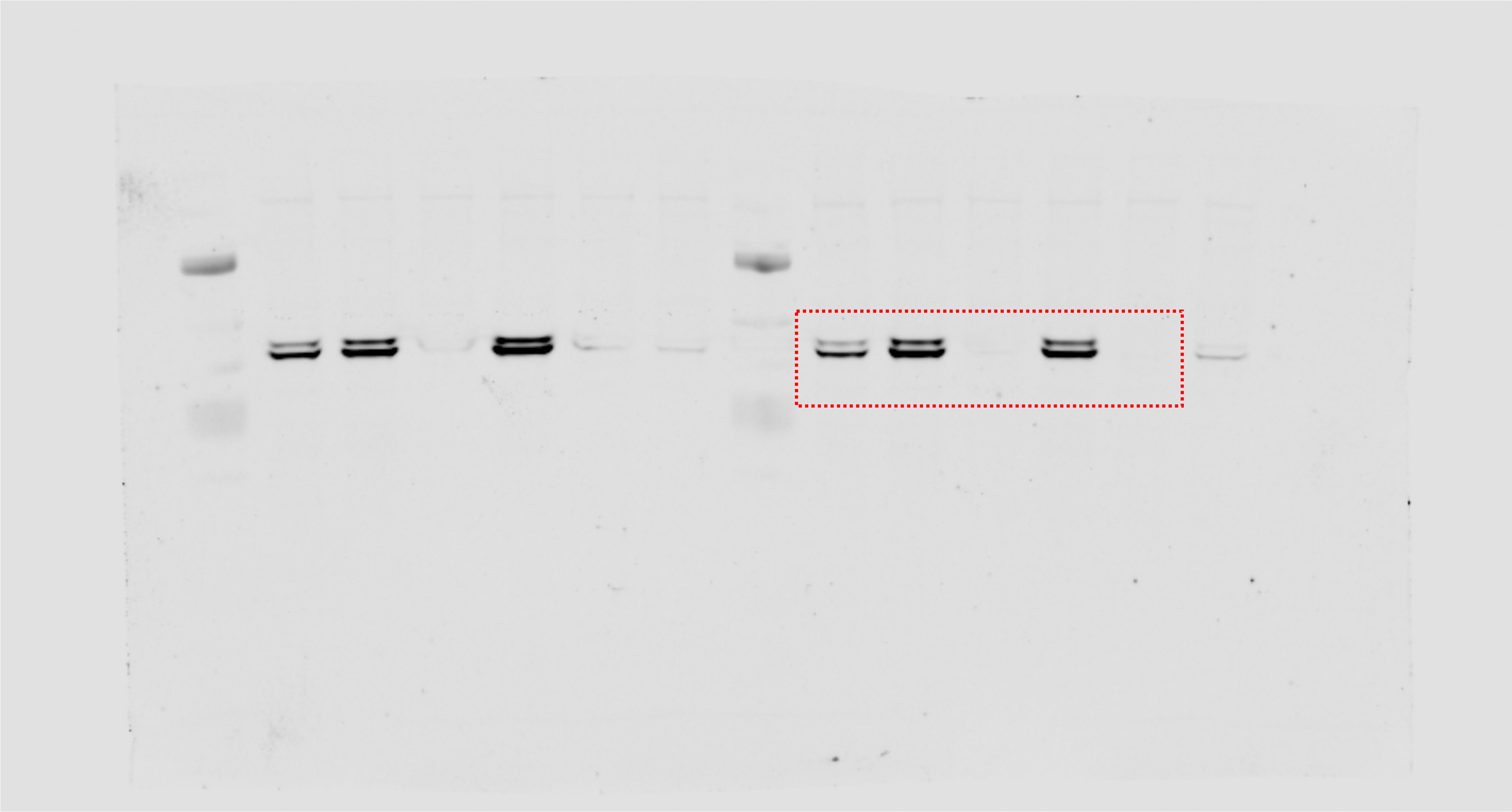

Figure 1A: ERK
Mouse anti-ERK1/2 (L34F12)
Cell Signaling #4696

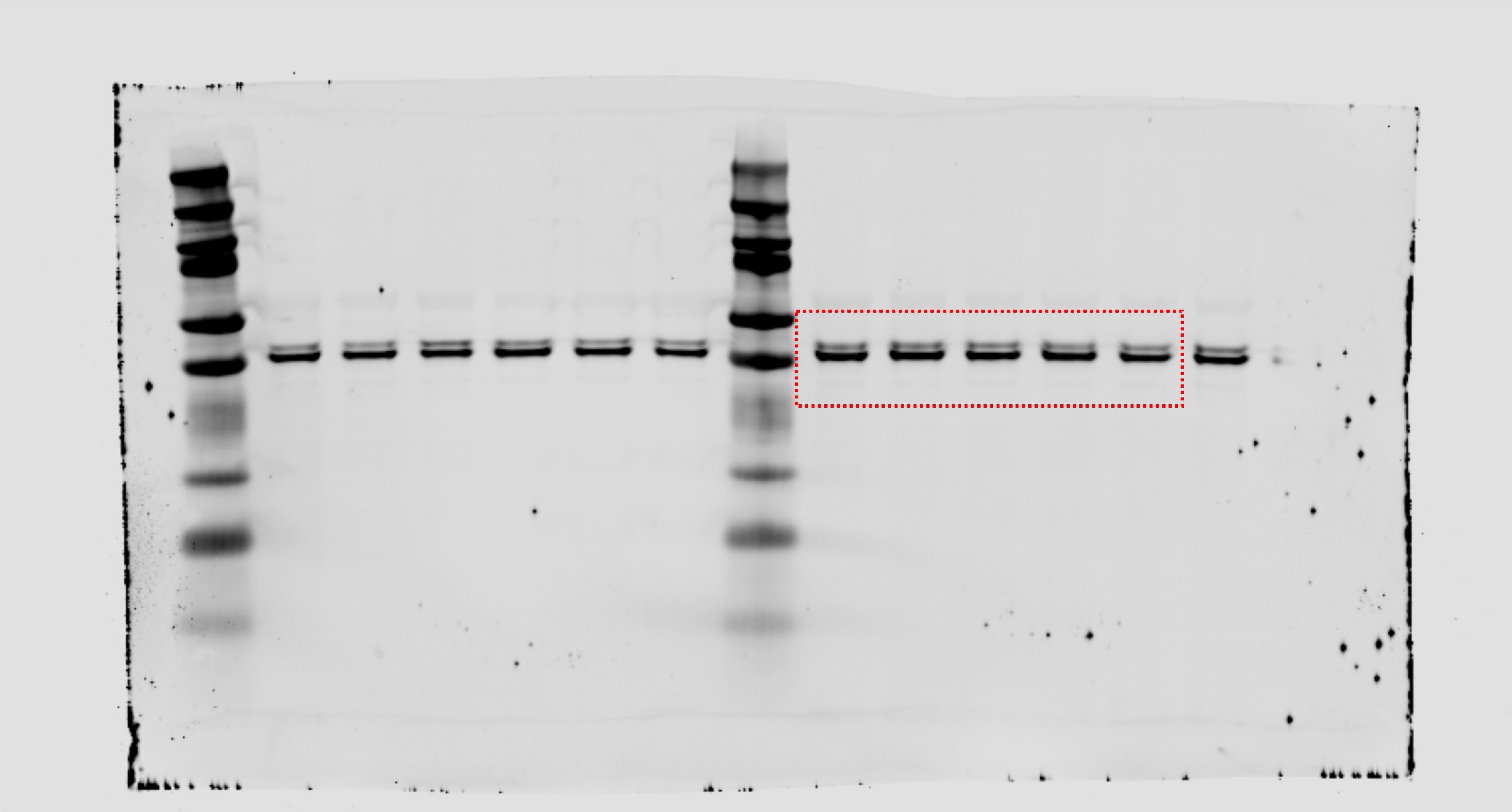

Figure 1A: GAPDH (re-probe of ERK blot)
Mouse anti-GAPDH
Santa Cruz #sc-47724

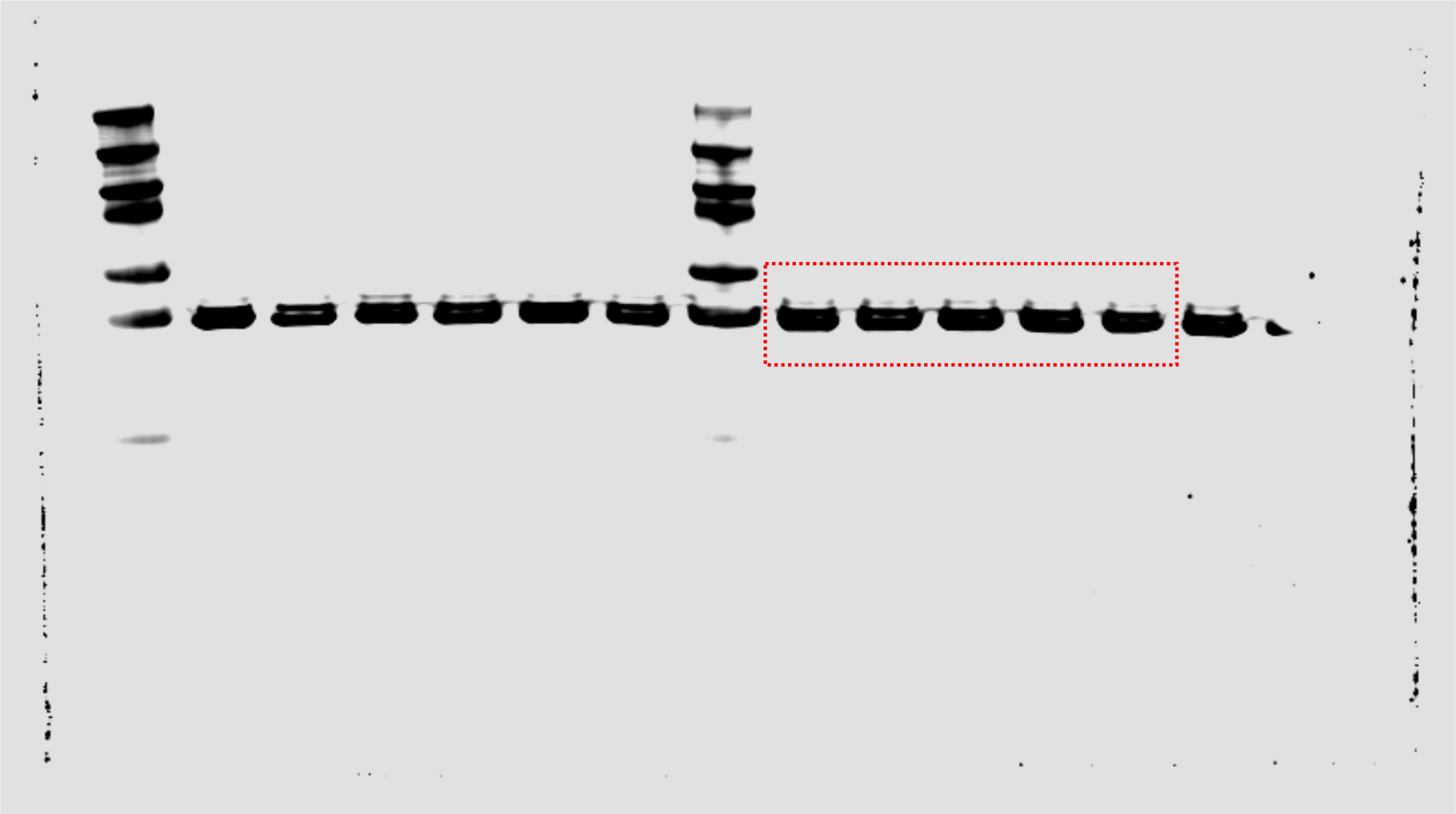

Figure 2A: Pan-Cadherin
Rabbit anti-Pan-Cadherin
Cell Signaling #4068

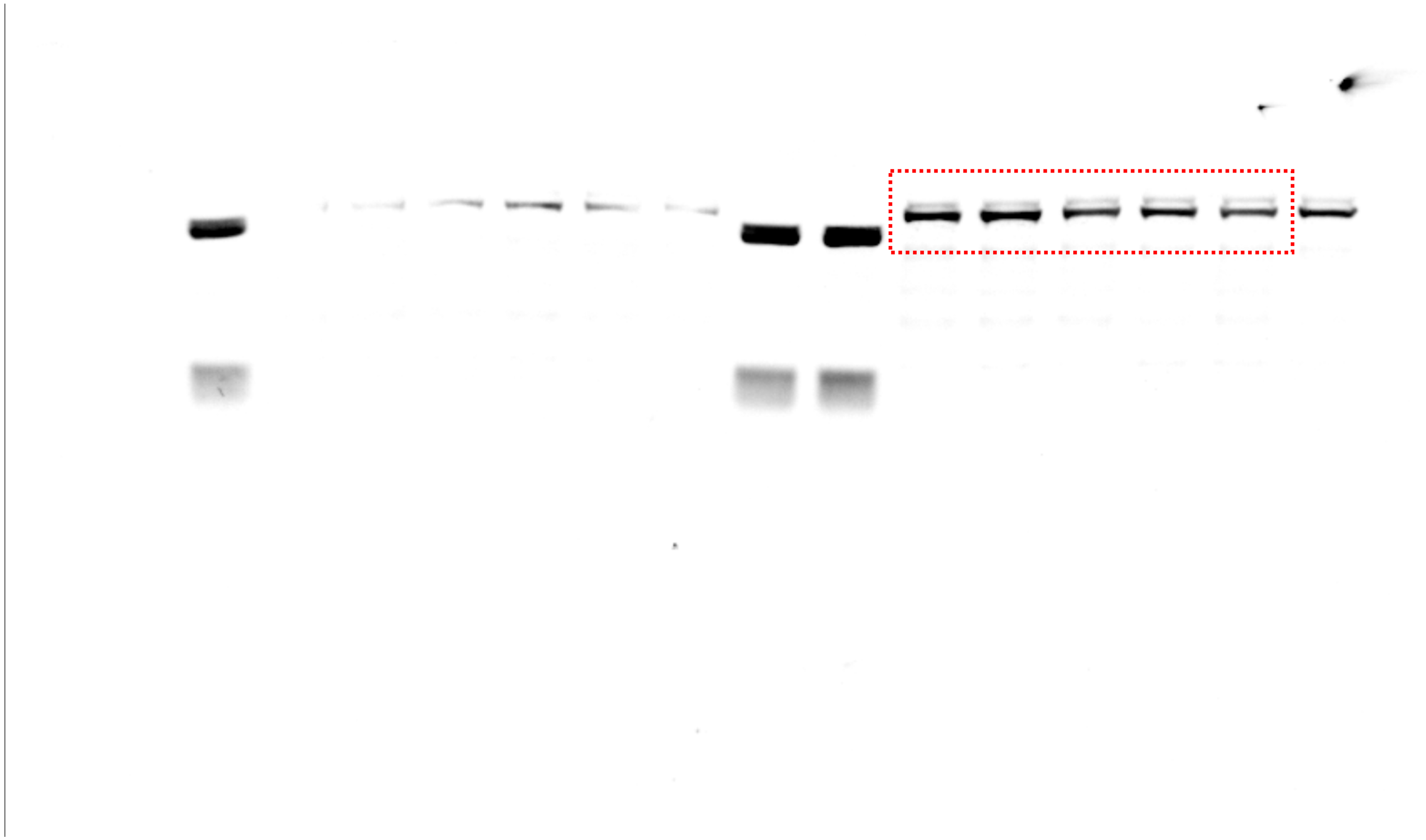

Figure 2A: DSG1
Mouse anti-Desmoglein 1
Santa Cruz #sc-137164

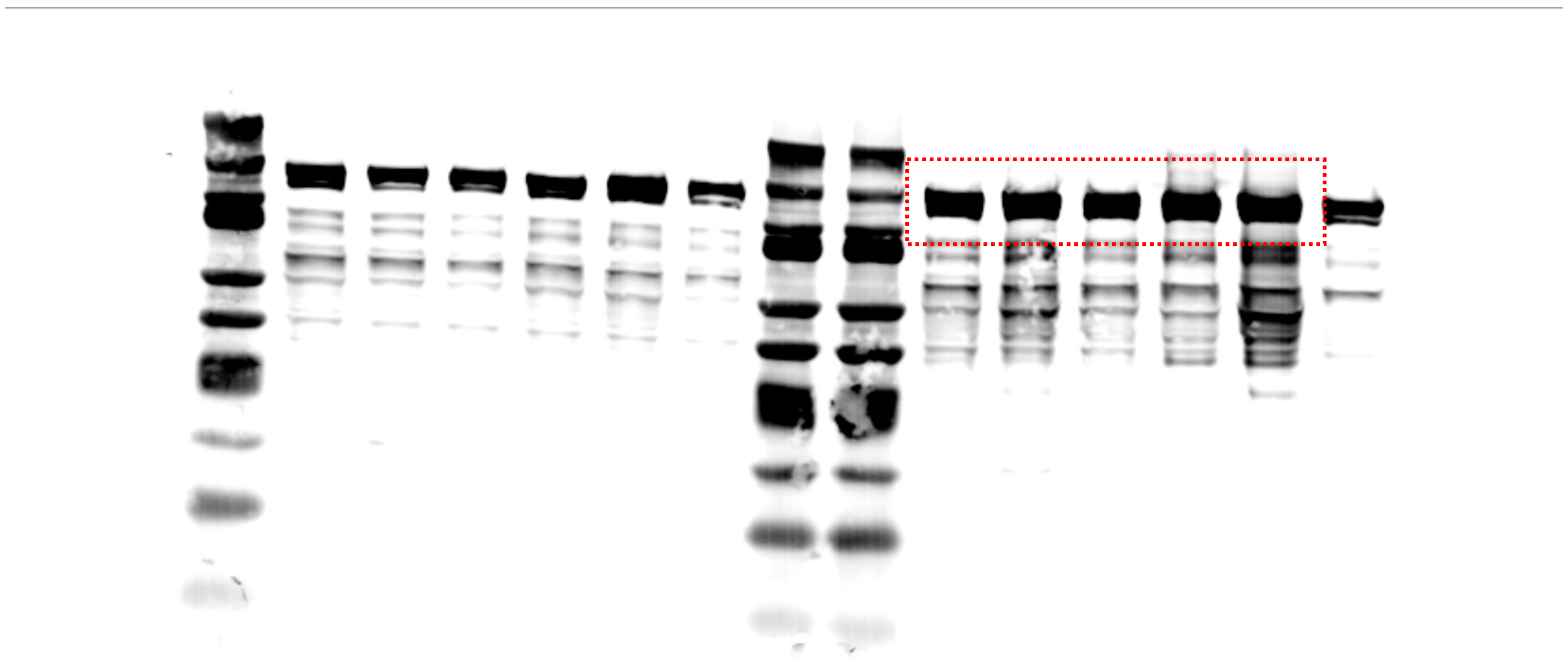

Figure 2A: DSG2
Mouse anti-Desmoglein 2
Santa Cruz #sc-80663

Figure 2A: β-Actin
Mouse anti-beta-Actin
Santa Cruz #sc- 47778

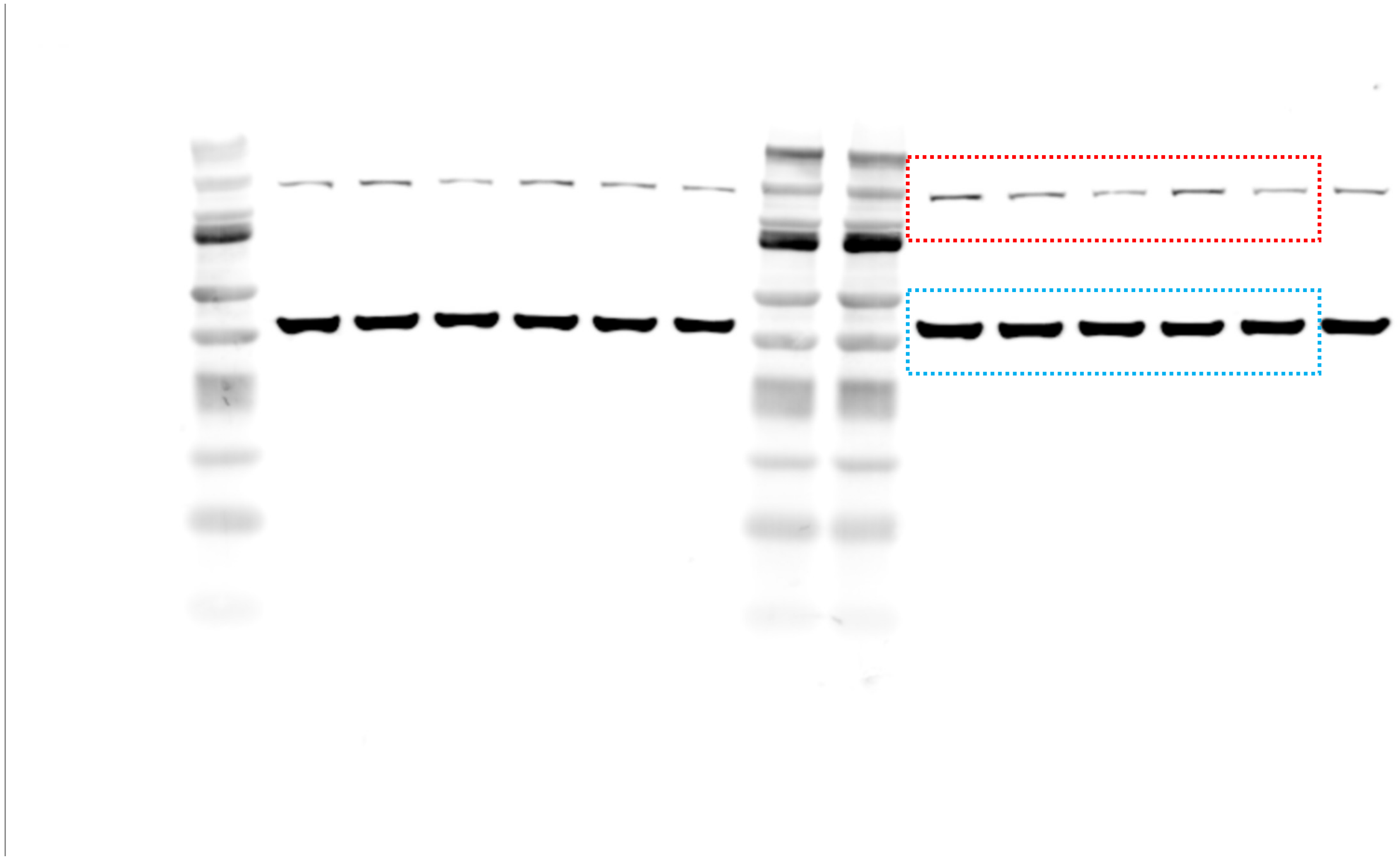

Figure 2A: DSG3
Mouse anti-Desmoglein 3
Santa Cruz #sc-53487

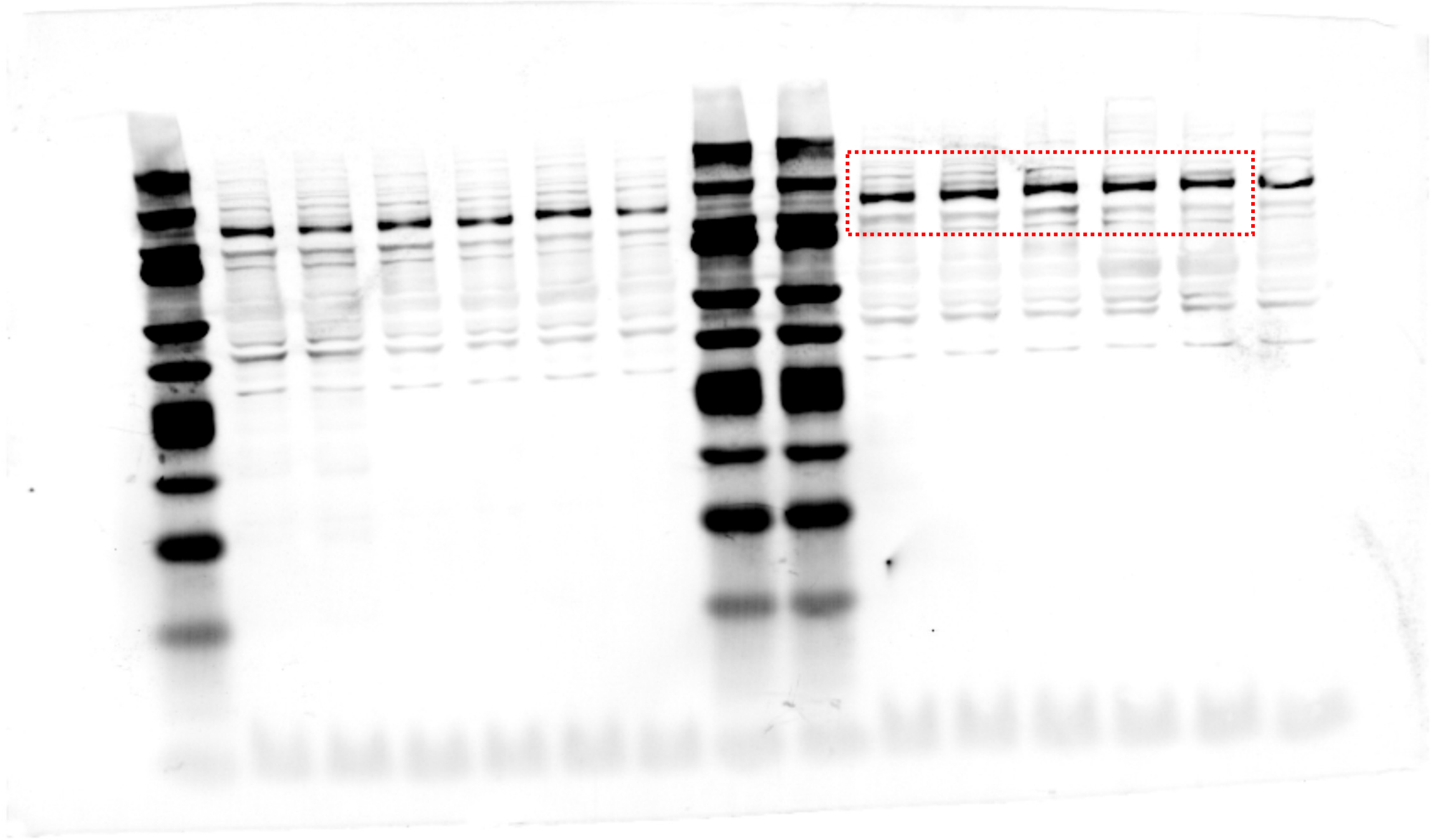

Figure 2A: Plakoglobin
Mouse anti-Gamma-catenin (plakoglobin)
Santa Cruz #sc-514115

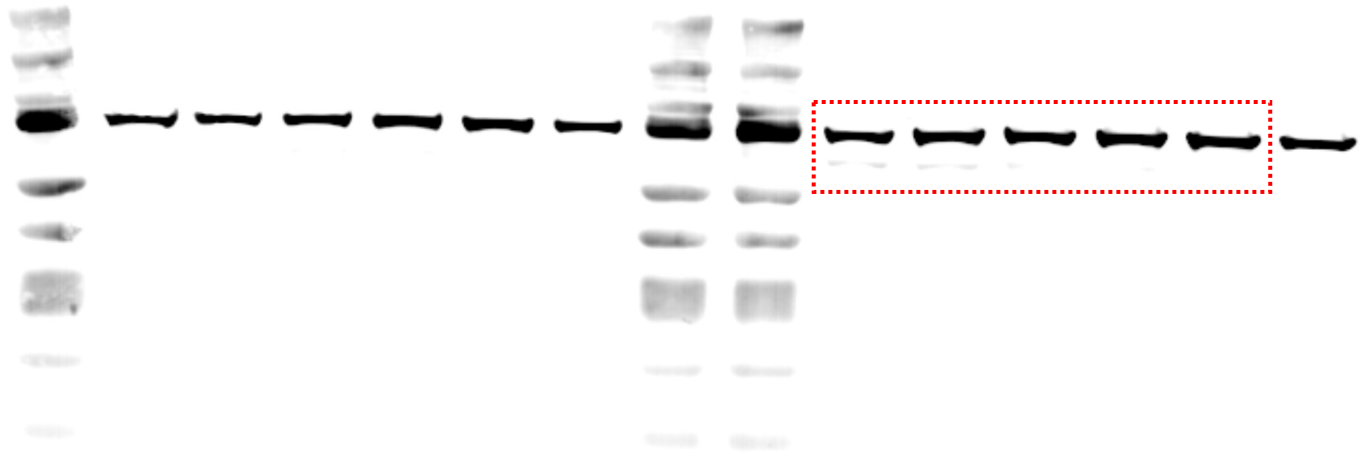

Supp Figure S1B: pERK
Rabbit anti-phospho-ERK1/2 (D13.14.4E)
Cell Signaling #4370

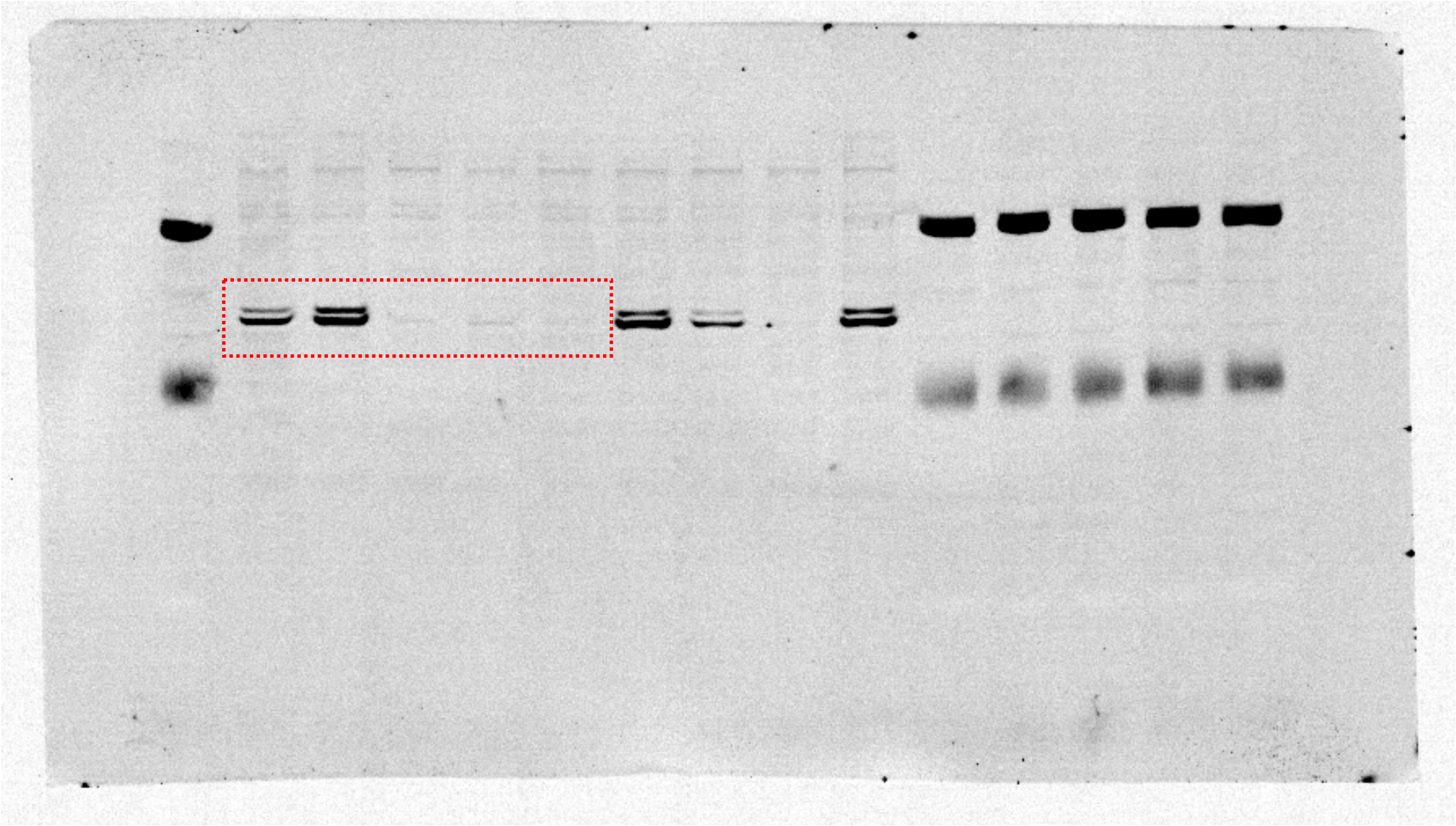

Supp Figure S1B: ERK
Mouse anti-ERK1/2 (L34F12)
Cell Signaling #4696

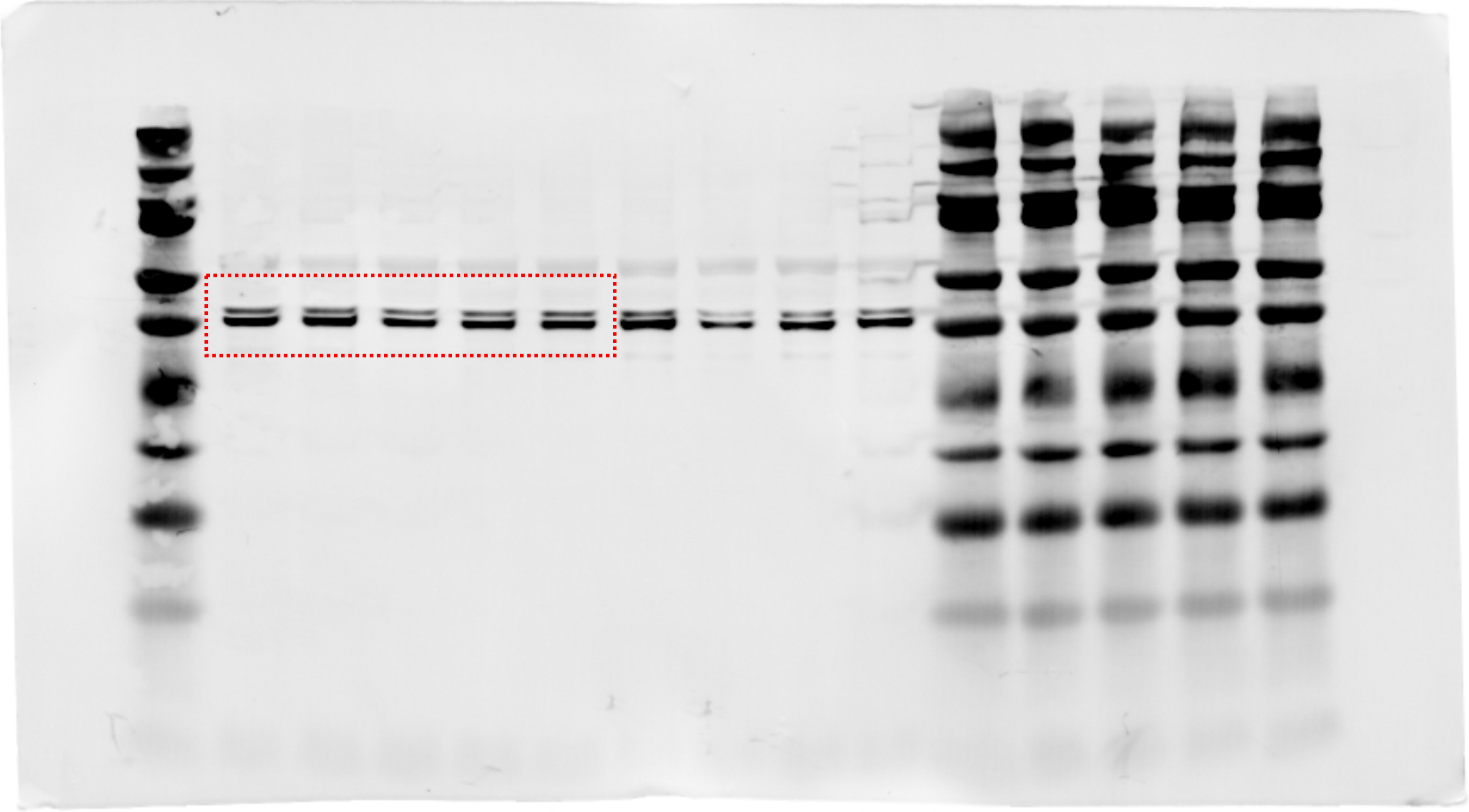

Supp Figure S1B: pERK
Rabbit anti-phospho-ERK1/2 (D13.14.4E)
Cell Signaling #4370

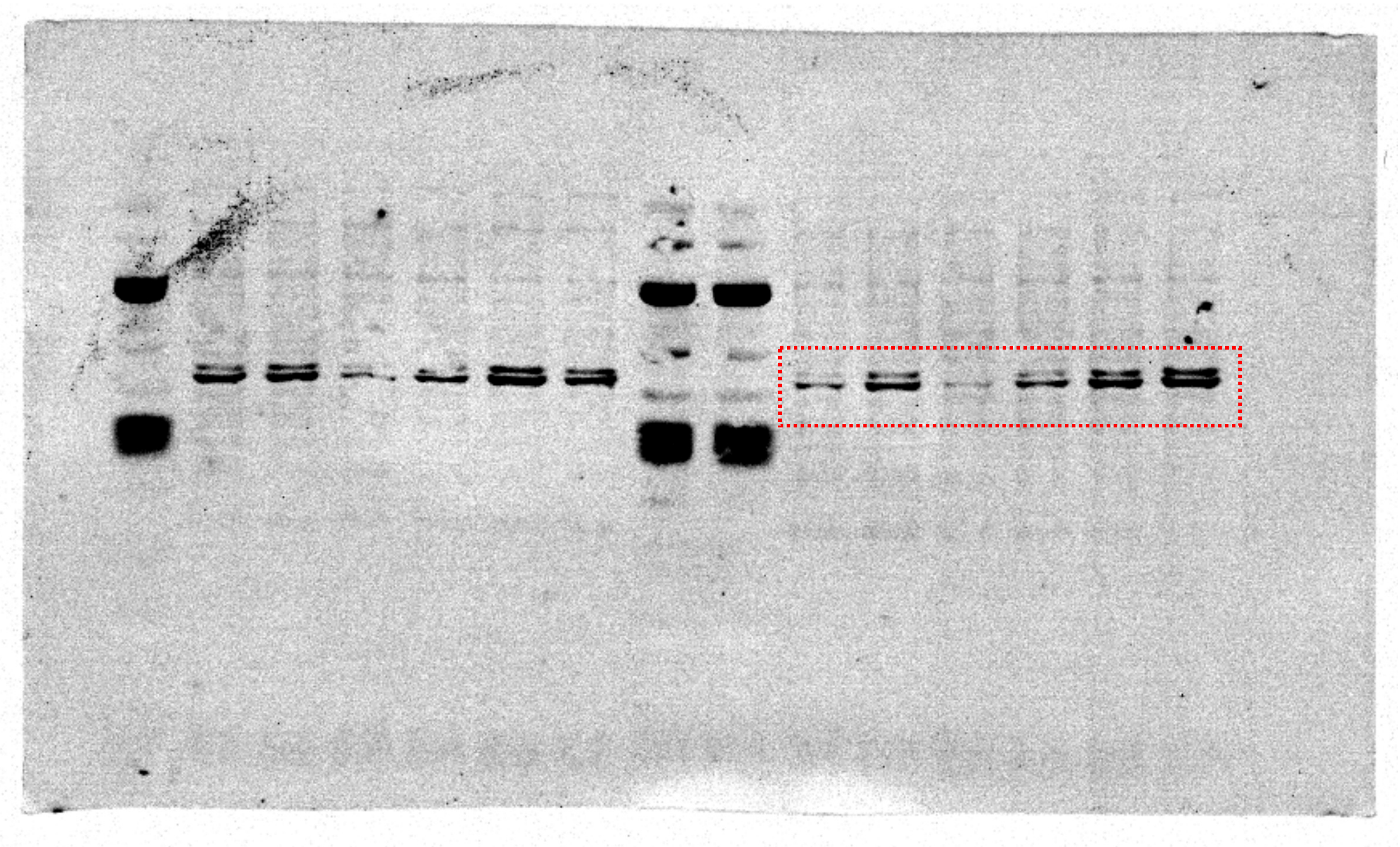

Supp Figure S1B: ERK
Mouse anti-ERK1/2 (L34F12)
Cell Signaling #4696

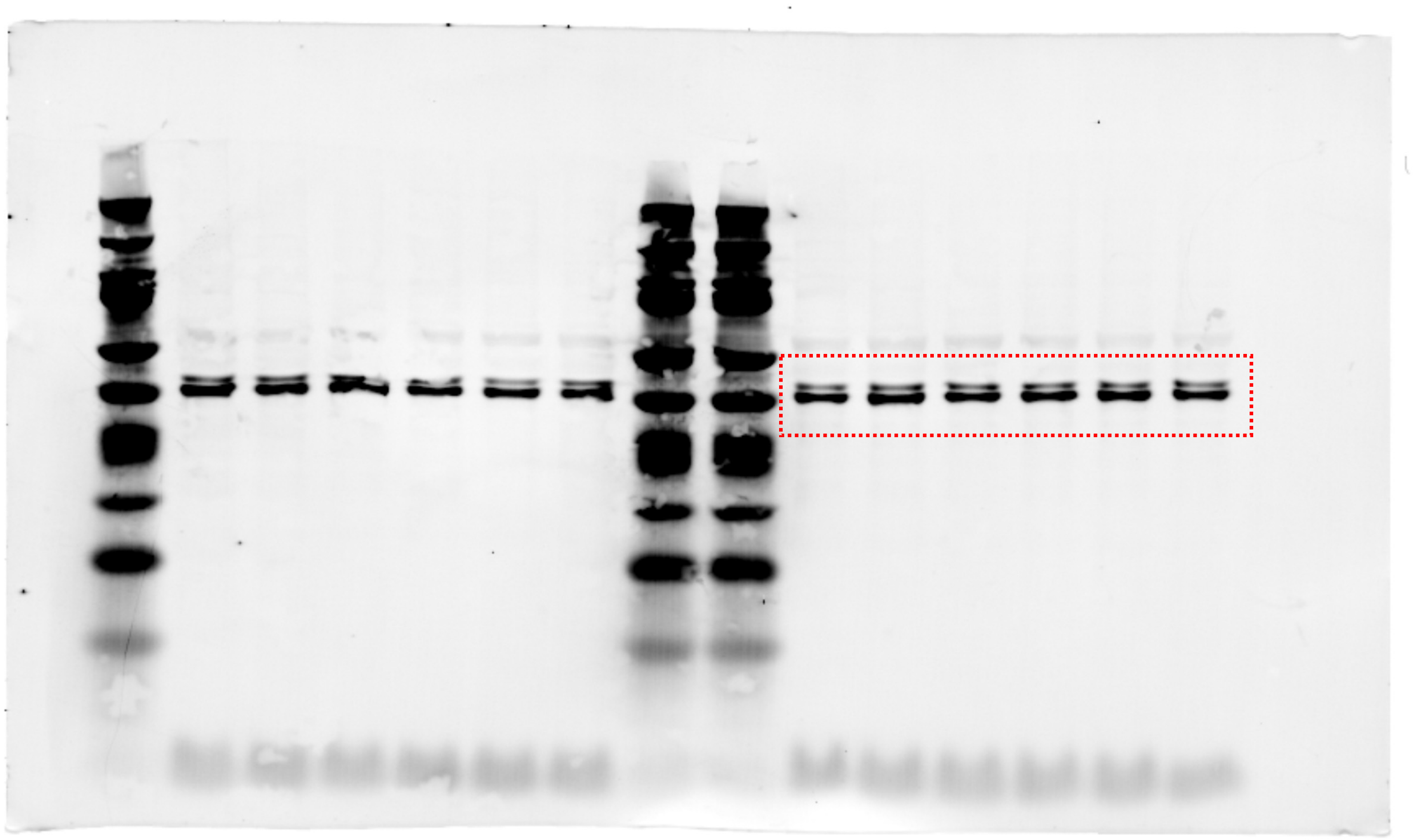

Supp Figure S2C: pERK
Rabbit anti-phospho-ERK1/2 (D13.14.4E)
Cell Signaling #4370

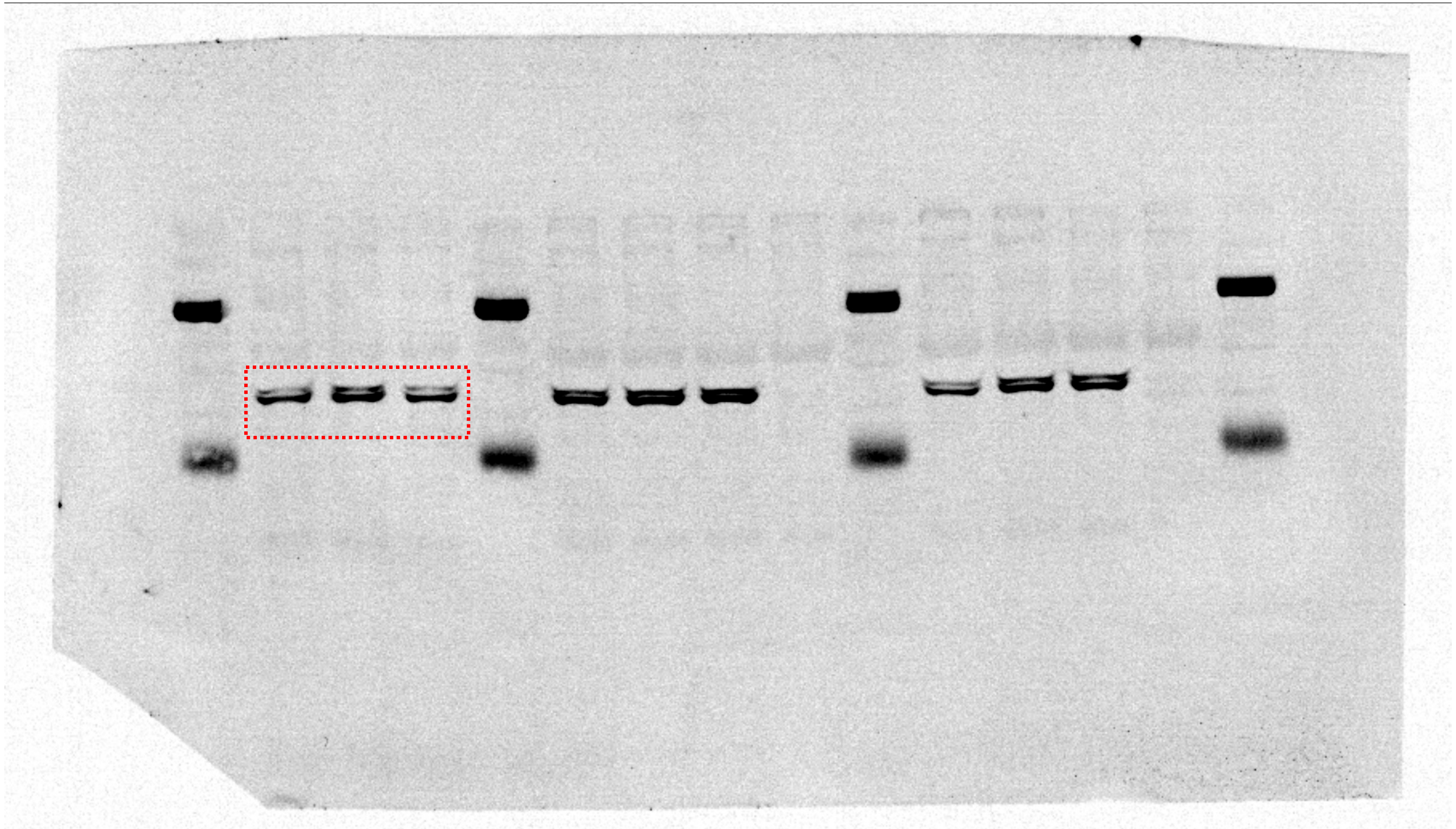

Supp Figure S2C: ERK
Mouse anti-ERK1/2 (L34F12)
Cell Signaling #4696

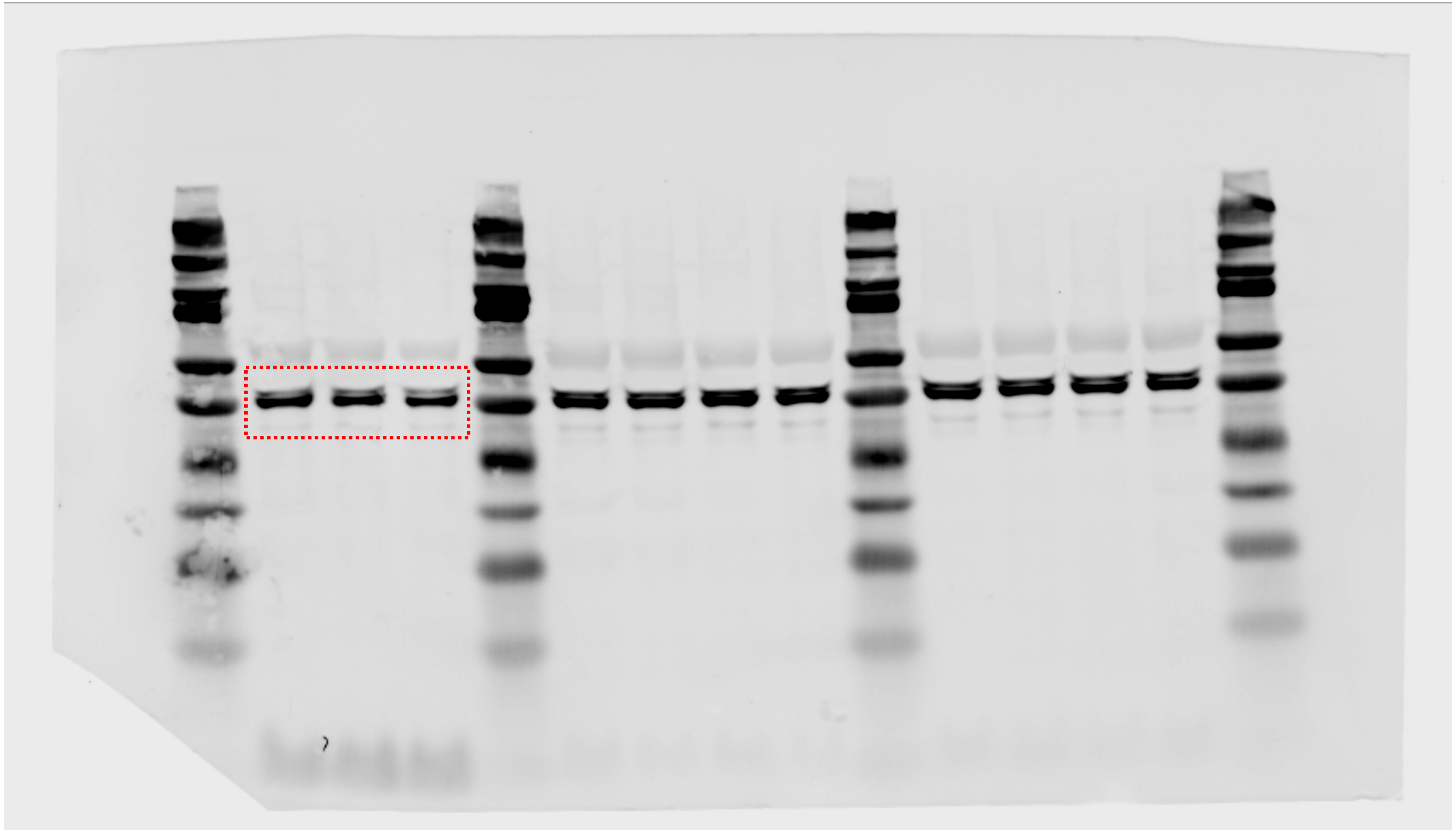

Supp Figure S2C: MEK
Mouse anti-MEK1/2 (L38C12)
Cell Signaling #4694

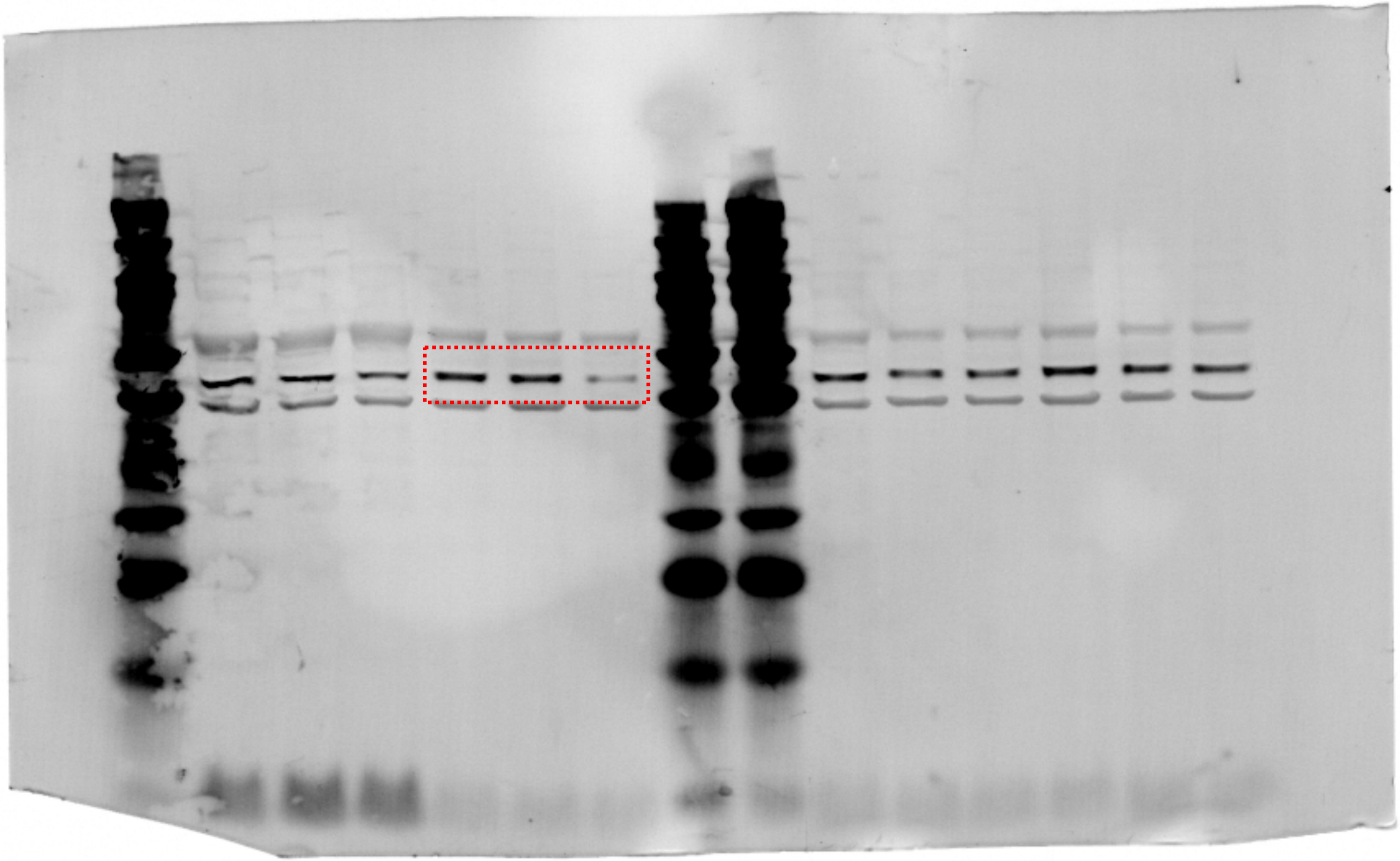

Supp Figure S2C: GAPDH
Rabbit anti-GAPDH (D16H11)
Cell Signaling #5174

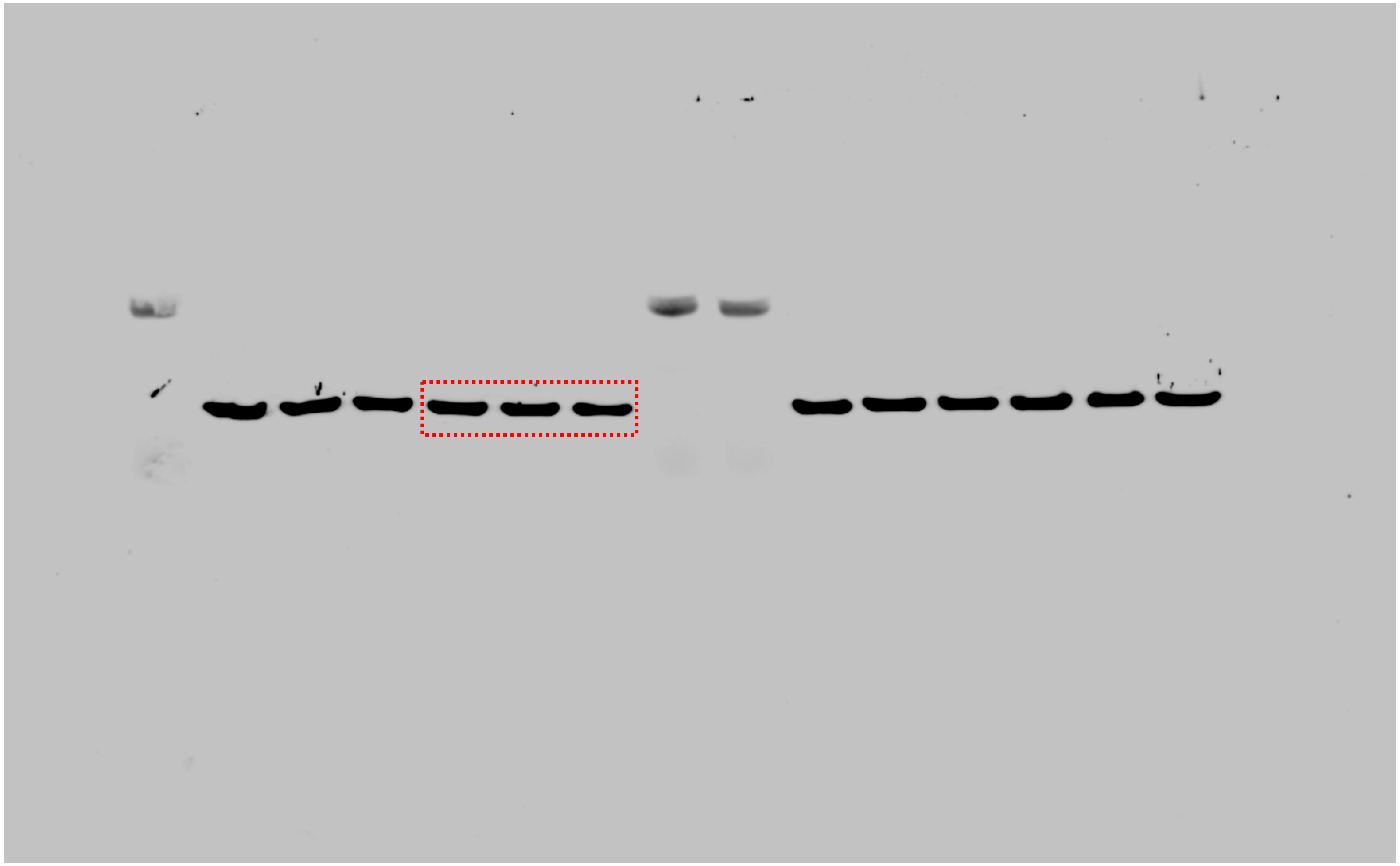

Supplement: Unedited blot and gel images [file jciinsight-9-182983-s033.pdf]
